# Small Airway Dysfunction Assessed by Impulse Oscillometry in Non-Asthmatic Children with Moderate-to-Severe Persistent Allergic Rhinitis During the Early Phase of Allergen Immunotherapy

**DOI:** 10.3390/jcm15145618

**Published:** 2026-07-17

**Authors:** Ercan Yılmaz, Erdem Topal

**Affiliations:** Department of Pediatric Allergy and Immunology, Faculty of Medicine, Inonu University, 44280 Malatya, Turkey; ercanyilmz44@gmail.com

**Keywords:** allergen immunotherapy, allergic rhinitis, impulse oscillometry, small airway dysfunction

## Abstract

**Background:** Allergic rhinitis (AR) is increasingly recognized as a systemic airway disease that may involve the lower respiratory tract even in the absence of overt asthma symptoms. Impulse oscillometry (IOS) is a sensitive and noninvasive method for detecting peripheral airway dysfunction in children. This cross-sectional study aimed to characterize peripheral airway function in non-asthmatic children with moderate-to-severe persistent allergic rhinitis during the early phase of allergen immunotherapy using impulse oscillometry. **Methods:** This cross-sectional case–control study included 69 children with moderate-to-severe persistent allergic rhinitis and 65 age- and sex-matched healthy controls. All patients had grass pollen sensitization and were receiving subcutaneous allergen immunotherapy. Children with clinical asthma symptoms, abnormal baseline spirometry, or chronic pulmonary disease were excluded. IOS parameters including R5, R20, R5–R20, X5, AX, and Fres were measured and compared between groups. **Results:** Demographic and anthropometric characteristics were similar between the groups. The allergic rhinitis group demonstrated significantly higher R5 values [0.41 (0.20) vs. 0.37 (0.18) kPa·L^−1^ s, *p* = 0.019], R5–R20 values [0.140 (0.13) vs. 0.10 (0.08) kPa·L^−1^ s, *p* = 0.001], and AX values [1.05 (1.14) vs. 0.86 (0.61) kPa·L^−1^, *p* = 0.035] compared with healthy controls. No significant differences were observed in R20, X5, X20, or Fres parameters. **Conclusions:** Children with moderate-to-severe persistent allergic rhinitis may exhibit subclinical small airway dysfunction despite the absence of asthma symptoms. IOS appears to be a useful tool for detecting early peripheral airway involvement and may help identify children at increased risk for future asthma development.

## 1. Introduction

Allergic rhinitis (AR) is one of the most common chronic inflammatory conditions in childhood and adolescence; it is characterized by symptoms such as nasal congestion, rhinorrhea, sneezing and nasal itching [1]. Allergic rhinitis, which has been reported to have seen a marked increase in prevalence worldwide in recent years, is not merely a localized disease affecting the upper airways; it can lead to a wide range of clinical problems, including impaired sleep quality, reduced academic performance, limitations in physical activity, and a decline in quality of life [2]. It is thought that chronic inflammation is more pronounced, particularly in cases of moderate-to-severe persistent allergic rhinitis, and may also affect the lower airways.

Although allergic rhinitis has traditionally been regarded as a disease of the upper airways, the ‘unified airway disease’ concept, which has been the subject of debate in recent years, has demonstrated that the upper and lower airways are closely interrelated from an anatomical, physiological and immunological perspective [3]. The relationship between allergic rhinitis and asthma has been demonstrated in numerous epidemiological and clinical studies. Whilst the vast majority of asthma patients have concomitant allergic rhinitis, it is also known that individuals with allergic rhinitis face an increased risk of developing asthma in later years [4,5]. Furthermore, it is noteworthy that bronchial hyperreactivity, eosinophilic airway inflammation and subclinical lower airway involvement can be detected even in some allergic rhinitis patients who do not report clinical asthma symptoms [6]. This suggests that inflammatory changes may be present in the lower airways, progressing silently, particularly during childhood before clinical asthma has developed.

Although spirometry is the most commonly used respiratory function test in clinical practice, it primarily assesses the large and central airways [7]. It is known that its sensitivity is limited, particularly when it comes to detecting early peripheral airway changes. Consequently, interest in more sensitive methods for assessing the small airways has grown in recent years.

The Impulse Oscillometry System (IOS) is a non-invasive, effort-independent method that assesses airway resistance and reactance during tidal breathing [8]. Due to its ease of use, particularly in the pediatric age group, IOS is being used increasingly frequently in pediatric allergy and pediatric respiratory practice. It has been reported that IOS can detect early-stage small airway dysfunction even in patients with normal spirometric values [9,10].

Although there are numerous studies on the use of IOS in children with asthma, the number of studies assessing small airway function in children diagnosed solely with allergic rhinitis and who do not report asthma symptoms is quite limited. In particular, as patients receiving allergen immunotherapy for moderate-to-severe persistent allergic rhinitis may represent a patient group with a higher inflammatory burden, investigating subclinical lower airway involvement in this population is of clinical importance. The detection of small airway dysfunction before the development of clinical asthma may provide guidance regarding the future respiratory risks of these patients. Furthermore, determining whether the early changes demonstrated by IOS are associated with the subsequent development of asthma may highlight the need for closer pulmonary monitoring in children with allergic rhinitis.

The aim of this cross-sectional study was to characterize small airway function using impulse oscillometry in non-asthmatic children with moderate-to-severe persistent allergic rhinitis who were in the early phase of allergen immunotherapy.

## 2. Materials and Methods

### 2.1. Study Design and Patient Population

This study was designed as an exploratory cross-sectional case–control study aimed at investigating lower airway involvement, conducted at a tertiary pediatric allergy clinic. The study included pediatric patients aged 6–18 years who had been diagnosed with moderate-to-severe persistent allergic rhinitis and were evaluated during the early phase of allergen immunotherapy.

The diagnosis of allergic rhinitis was established based on clinical symptoms and demonstrated sensitivity to aeroallergens, in accordance with the criteria of the Allergic Rhinitis and its Impact on Asthma (ARIA) guidelines [1]. The definition of moderate-to-severe persistent allergic rhinitis was based on impairment of daily living activities, sleep disturbance, reduced school performance and the presence of persistent symptoms.

All patients included in the study had pollen sensitivity and were those who had commenced subcutaneous allergen immunotherapy (SCIT) containing a grass pollen mixture. In all cases, immunotherapy was administered using the Roxall^®^ GrassMix preparation (ROXALL Medizin GmbH, Hamburg, Germany). The patients were being followed up at the pediatric allergy and immunology outpatient clinic with a diagnosis of moderate-to-severe persistent allergic rhinitis and comprised individuals in whom pollen sensitivity had been confirmed based on clinical history, skin prick testing and/or serum-specific IgE results. The control group consisted of healthy children who were similar to the study group in terms of age and gender, and who had no history of chronic respiratory diseases, allergic diseases, signs of active infection, or regular medication use. All participants in the control group were included in the study following a detailed clinical assessment.

Patients were screened for asthma symptoms, including recurrent wheezing, breathlessness on exertion, night-time cough, episodic dyspnea, and a history of a previous asthma diagnosis by a doctor. Patients with a diagnosis of asthma, those describing asthma symptoms, those with chronic lung disease, congenital heart disease, primary immunodeficiency, systemic inflammatory disease, a respiratory tract infection within the previous four weeks, active smoking, or those unable to comply with IOS measurements were excluded from the study. Furthermore, all patients had undergone a baseline spirometry examination as part of routine clinical assessment prior to the initiation of allergen immunotherapy. Patients with a forced expiratory volume in the first second (FEV_1_) value below 80% of the predicted value, suggesting large airway obstruction, or a mid-expiratory flow rate (FEF25–75) value below 70% of the predicted value, suggesting small airway obstruction, were excluded from the study.

### 2.2. Clinical and Laboratory Assessment

Detailed demographic and clinical characteristics of all participants were recorded. Age, gender, exposure to smoking, concomitant atopic diseases, family history of atopic diseases, history of breastfeeding, and pet ownership were assessed. Patients’ height and weight measurements were taken using standard methods, and height z-scores and weight z-scores were calculated according to age and gender. Anthropometric assessments were performed in accordance with national reference data [11]. For the assessment of allergic rhinitis symptom severity, a visual analogue scale (VAS) was used to evaluate nasal and ocular symptoms prior to immunotherapy. VAS scores were assessed on a scale of 0–10.

Laboratory data for all patients were obtained retrospectively from medical records. Peripheral blood eosinophil counts were recorded from complete blood count analyses, and total serum immunoglobulin E (IgE) levels were evaluated based on patients’ laboratory files and hospital records. Aeroallergen sensitivity was assessed using skin prick tests and/or serum-specific IgE measurements.

### 2.3. Assessment of Airway Functions Using Impulse Oscillometry System

Airway resistance and reactance were assessed using an impulse oscillometry system (IOS) (Jaeger MasterScreen IOS, CareFusion, Yorba Linda, CA, USA). The device was calibrated daily prior to testing using a standard 3 L syringe and reference resistance, in accordance with the manufacturer’s instructions. All measurements were performed in accordance with the technical standards for respiratory oscillometry published by the European Respiratory Society (ERS) and supported by the American Thoracic Society [12]. Participants were instructed to avoid heavy meals, vigorous exercise, and short-acting bronchodilators for at least 4–6 h prior to testing. Measurements were conducted in a quiet environment with participants in a seated position, wearing a nose clip and maintaining a neutral head position. To minimize upper airway artifact, participants firmly supported their cheeks and the floor of the mouth with their hands.

During the procedure, subjects were asked to breathe normally (tidal breathing) through a mouthpiece connected to the IOS device for approximately 30–45 s, ensuring a tight seal around the mouthpiece. At least three technically acceptable measurements were obtained from each participant. Measurements were considered acceptable if they met the following criteria: absence of artifacts such as coughing, swallowing, vocalization, air leakage, or irregular breathing; a stable tidal breathing pattern; and coherence values of ≥0.80 at 5 Hz and ≥0.90 at frequencies ≥10 Hz. The mean values of the acceptable measurements were used for analysis.

The IOS parameters evaluated included total airway resistance at 5 Hz (R5), central airway resistance at 20 Hz (R20), and the difference between R5 and R20 (R5–R20), which reflects peripheral (small airway) resistance. Reactance parameters included reactance at 5 Hz (X5), reactance area (AX), and resonance frequency (Fres). Negative X5 values indicate reduced elastic recoil and peripheral airway involvement.

All IOS parameters were expressed as absolute values and, where available, as percentages of predicted values generated by the IOS software using age-, sex-, height-, and ethnicity-specific reference equations. The primary objective of the study was to compare continuous IOS parameters between children with moderate-to-severe persistent allergic rhinitis and age- and sex-matched healthy controls. Accordingly, R5–R20, AX, and Fres were analyzed as continuous indicators of peripheral airway function, and the interpretation of airway involvement was based on comparisons with the matched healthy control group rather than on a predefined categorical classification of small airway dysfunction.

### 2.4. Sample Size and Power Analysis

The study was designed as a cross-sectional pilot study aimed at assessing small airway dysfunction in children with moderate-to-severe persistent allergic rhinitis using impulse oscillometry. It was noted that the prevalence of allergic rhinitis in childhood is reported in the literature to be approximately 20–30%. It was also assumed that the prevalence of subclinical small airway dysfunction in children with allergic rhinitis might be around 20%. In the sample size calculation, an alpha level of 0.05, a beta level of 0.20 and a statistical power of 80% were assumed. Based on a power analysis assuming an expected rate of small airway dysfunction of 20%, the minimum sample size required for the study was calculated to be approximately 60 patients.

### 2.5. Statistical Analysis

Statistical analysis was performed using SPSS Statistics (v.21.0; IBM Corp., Armonk, NY, USA). Normality was assessed using the Kolmogorov–Smirnov test. Descriptive statistics were expressed as frequency and percentage for categorical variables whereas the median (IQR or min–max) was used for quantitative data. Categorical variables were compared using the Pearson’s chi-square test or Fisher’s exact test, and quantitative variables were compared using the Mann–Whitney U test. Two-tailed *p*-values < 0.05 were considered statistically significant.

## 3. Results

### 3.1. Demographic, Clinical and Laboratory Characteristics

A total of 69 children diagnosed with moderate-to-severe persistent allergic rhinitis were included in the study. Thirty-seven of the patients were boys (53.6%) and 32 were girls (46.4%). The median age of the participants was 13 years (range 6–18 years). The median age at diagnosis was 8 years (range 4–11 years) and the median duration of symptoms was 6 years (range 3–10 years). Exposure to smoking was present in 15 patients (21.7%). A history of breastfeeding for more than six months was present in 61 patients (88.4%). The presence of pets in the home was noted in 9 patients (13%). When personal history of atopic diseases was assessed, 52 patients (75.3%) had a history of allergic conjunctivitis, 9 patients (13%) had a history of eczema, 2 patients (2.9%) had a history of drug allergy, and 1 patient (1.4%) had a history of food allergy. Upon examination of family history, a history of asthma was found in 7 patients (10.1%), allergic rhinitis in 17 patients (24.6%), and eczema in 2 patients (2.9%). In the symptom assessment, the median VAS score for nasal symptoms prior to immunotherapy was 8 (5–10), whilst the median VAS score for ocular symptoms was 7 (3–10). Laboratory investigations revealed a median total IgE level of 599 IU/mL (17–2000 IU/mL), a median eosinophil count of 1.8/mm^3^ (1.31–6.1/mm^3^) and a median eosinophil percentage of 3.1% (1.5–14.1%). When assessing sensitivity to airborne allergens, pollen sensitivity was present in all patients and was detected in all 69 patients (100%). In addition, 12 patients (17.3%) were sensitive to house dust mites, 9 patients (13%) to mold, and 13 patients (18.8%) to cat dander (Table 1).

When the allergic rhinitis group was compared with the healthy control group, there were 37 male patients (53.6%) in the allergic rhinitis group and 32 male individuals (49.2%) in the control group; no significant difference was observed in terms of gender distribution (*p* = 0.61). The median age was 13 years (6–18 years) in the allergic rhinitis group and 15 years (5–17 years) in the control group; no significant difference was observed in terms of age (*p* = 0.144). Exposure to tobacco smoke was present in 18 individuals (26.5%) in the allergic rhinitis group and 14 individuals (21.5%) in the control group; no significant difference was found between the two groups (*p* = 0.50). Anthropometric measurements were also comparable between the groups. Height z-scores were −0.04 ± 1.86 in the allergic rhinitis group and 0.14 ± 1.92 in the control group (*p* = 0.708), whilst weight z-scores were 0.30 ± 1.31 and 0.35 ± 1.24, respectively (*p* = 0.659) (Table 2).

### 3.2. Impulse Oscillometry Findings

Upon examination of the pulse oscillometry parameters, the R5 value was found to be significantly higher in the allergic rhinitis group compared to the control group [0.41 (0.20) kPa·L^−1^ s vs. 0.37 (0.18) kPa·L^−1^ s, *p* = 0.019]. Similarly, the R5–R20 value, which indicates small airway resistance, was also significantly higher in the allergic rhinitis group [0.140 (0.13) kPa·L^−1^ s vs. 0.10 (0.08) kPa·L^−1^ s, *p* = 0.001]. The AX value was also found to be higher in the allergic rhinitis group [1.05 (1.14) kPa·L^−1^ vs. 0.86 (0.61) kPa·L^−1^, *p* = 0.035]. In contrast, there was no significant difference in the X20 value when compared with the control group [0.02 (0.15) kPa·L^−1^ s vs. 0.01 (0.09) kPa·L^−1^ s, *p* = 0.570]. R20 values were 0.25 (0.11) kPa·L^−1^ s in the allergic rhinitis group and 0.25 (0.13) kPa·L^−1^ s in the control group; no significant difference was observed between the groups (*p* = 0.956). Similarly, X5 values were −0.10 (0.07) kPa·L^−1^ s and −0.09 (0.07) kPa·L^−1^ s, respectively, and no significant difference was detected (*p* = 0.101). Fres values were found to be 18.58 (4.79) Hz in the allergic rhinitis group and 19.14 (8.22) Hz in the control group, and no significant difference was observed between the groups (*p* = 0.312) (Table 2).

## 4. Discussion

In this study, impulse oscillometry parameters were assessed in non-asthmatic children with moderate-to-severe persistent allergic rhinitis during the early phase of allergen immunotherapy and compared with those of age- and sex-matched healthy controls. The main finding of our study was that the R5, R5–R20 and AX parameters, which particularly reflect small airway function, were found to be significantly higher in the moderate-to-severe persistent allergic rhinitis group. In contrast, no significant difference was detected in the R20 parameter, which reflects central airway resistance. These findings suggest that subclinical small airway dysfunction may be present in children with moderate-to-severe persistent allergic rhinitis who do not clinically report asthma symptoms.

The close relationship between allergic rhinitis and asthma has long been recognized and is best explained by the concept of the unified airway, which considers the upper and lower airways as a single functional and immunological unit [3,13]. Both compartments share a common embryological origin, similar epithelial architecture, and overlapping inflammatory pathways, suggesting that allergic inflammation represents a systemic process affecting the entire respiratory tract rather than two isolated diseases [14]. Type 2 immune responses, characterized by eosinophilic inflammation, epithelial barrier dysfunction, and the production of cytokines such as IL-4, IL-5, and IL-13, have been demonstrated in both the nasal and bronchial mucosa, supporting the biological basis of this airway continuum [15]. Within this framework, persistent type 2 inflammation may extend beyond the upper airway through circulating inflammatory mediators, activated inflammatory cells, and shared mucosal immune responses, promoting epithelial injury, mucus hypersecretion, airway wall edema, and early structural changes in the peripheral airways. These pathological alterations may increase peripheral airway resistance while conventional spirometric indices remain within the normal range, providing a plausible explanation for the IOS abnormalities observed in our cohort. Similar findings have been reported in Asian pediatric populations, where impulse oscillometry detected evidence of peripheral airway dysfunction in children with allergic rhinitis despite normal spirometry and the absence of clinically diagnosed asthma, further supporting the concept that small airway involvement may represent an early manifestation of lower airway disease [16,17].

There are studies in the literature suggesting that inflammatory changes in the lower airways may begin before clinical asthma develops in individuals with allergic rhinitis. Saranz RJ et al. reported that bronchial function abnormalities were detected in children with allergic rhinitis who did not have a diagnosis of asthma [18]. Similarly, it has been reported that findings such as bronchial hyperreactivity, increased exhaled nitric oxide levels and subclinical airway inflammation are also more common in children with allergic rhinitis [5]. In our study, the detection of significant deterioration in IOS parameters, particularly those indicating small airway resistance, supports the possibility that subclinical lower airway involvement may be present in these patients, even though it has not yet become clinically apparent.

Impulse oscillometry is an effort-independent method that assesses airway resistance and reactance during tidal breathing, offering significant advantages particularly in the pediatric age group. As it requires less cooperation than spirometry and can assess the peripheral airways more sensitively, its use in pediatric allergy and pediatric respiratory practice has increased in recent years [19]. In particular, the R5–R20 parameter is recognized as an important indicator of peripheral airway resistance and is considered one of the early markers of small airway dysfunction [20]. In our study, the finding that the R5–R20 value was significantly higher in the allergic rhinitis group indicates an increase in resistance at the small airway level. Similarly, the increase in the AX value also suggests impaired elastic properties in the peripheral airways and ventilation heterogeneity [9]. Conversely, the absence of significant differences in R20 values supports the notion that the central airways are relatively preserved, while early-stage functional changes are primarily concentrated in the distal airways. This finding is consistent with the description of small airways as a ‘silent zone’ in the early stages of obstructive airway diseases [21].

The fact that all patients in our study had commenced allergen immunotherapy due to moderate-to-severe persistent allergic rhinitis suggests that the patient group in the study represents a specific phenotype carrying a higher inflammatory burden. It has been suggested that severe and persistent allergic inflammation may not be limited to the upper airways but may also affect the peripheral airways [22]. Therefore, performing small airway assessment using IOS in children receiving immunotherapy may be clinically valuable.

It is known that allergen immunotherapy can have beneficial effects on both the upper and lower airways by suppressing allergic inflammation [23]. Therefore, monitoring small airway dysfunction detected by IOS throughout the course of immunotherapy may contribute to an objective assessment of the response to treatment. Future prospective longitudinal studies may better elucidate the long-term effects of immunotherapy on peripheral airway function.

This study has several limitations. First, it was conducted at a single tertiary center and included only non-asthmatic children with moderate-to-severe persistent allergic rhinitis who were predominantly sensitized to grass pollen and evaluated at the initiation of subcutaneous allergen immunotherapy. Because the number of children primarily sensitized to perennial allergens was limited, we focused on this relatively homogeneous population to minimize potential confounding related to different sensitization profiles, although this may limit the generalizability of our findings. Second, the cross-sectional design precludes conclusions regarding causality or the prognostic significance of the observed IOS abnormalities, and longitudinal changes in peripheral airway function during immunotherapy could not be assessed. Third, children with impaired baseline spirometry were excluded to specifically investigate whether IOS could detect peripheral airway dysfunction despite preserved conventional spirometry. Consequently, our cohort represents a relatively healthier subgroup, and the prevalence of lower airway involvement may have been underestimated. In addition, asymptomatic bronchial hyperresponsiveness cannot be completely excluded because bronchial provocation and bronchodilator reversibility testing were not performed. Despite these limitations, the inclusion of an age- and sex-matched healthy control group and the assessment of peripheral airway function using impulse oscillometry in non-asthmatic children with moderate-to-severe allergic rhinitis provide important insights into early lower airway involvement. Prospective multicenter longitudinal studies are warranted to determine the clinical significance of these findings and their relationship with future asthma development.

## 5. Conclusions

In this study, impulse oscillometry detected evidence of peripheral airway dysfunction in non-asthmatic children with moderate-to-severe persistent allergic rhinitis. In particular, the increased R5–R20 and AX values suggest that small airway involvement may be present despite preserved conventional spirometric function and the absence of clinical asthma symptoms. These findings support the concept that lower airway involvement may occur early in the course of allergic rhinitis, consistent with the unified airway hypothesis. As a non-invasive, effort-independent, and sensitive technique, impulse oscillometry may represent a useful adjunctive tool for the assessment of peripheral airway function in this patient population. Further prospective longitudinal studies are needed to determine the clinical significance of these abnormalities, their persistence over time, their response to allergen immunotherapy, and their potential association with subsequent asthma development.

## Figures and Tables

**Table 1:** Demographic and Laboratory Characteristics of Participants (*n* = 69).

Category	Variable	*n* (%)
Demographics	Male sex	37 (53.6)
	Age, years, median (min–max), years	13 (6–18)
	Age of diagnosis, median (min–max), years	8 (4–11)
	Symptom duration, median (min–max), years	6 (3–10)
	Smoking exposure	15 (21.7)
	Breast feed > 6 month	61 (88.4)
	Pet in house	9 (13)
Personal history of atopy	Allergic conjunctivitis	52 (75.3)
	Eczema history	6 (8.6)
	Drug allergy history	2 (2.9)
	Food allergy history	1 (1.4)
Family history of atopy	Asthma	7 (10.1)
	Allergic rhinitis	17 (24.6)
	Eczema	2 (2.9)
Symptom score	VAS score before AIT, for rhinitis, median (min–max)	8 (5–10)
	VAS score before AIT, for eyes, median (min–max),	7 (3–10)
Laboratory	Total Ig E, median (min–max), IU/mL	599 (17–2000)
	Eosinophil count/mm^3^, median (min–max)	1.8 (1.31–6.1)
	Eosinophil percent, median (min–max), %	3.1 (1.5–14.1)
Aeroallergen sensitization	Pollen	69 (100)
	House dust mite	12 (17.3)
	Mold	9 (13)
	Cat dander	13 (18.8)

VAS: Visual analogue scale, AIT: Allergen immunotherapy.

**Table 2:** Comparison of Oscillometric Measurements Between the Allergic Rhinitis and Healthy Control Groups.

Variable	Allergic Rhinitis Group (*n*: 69)	Healthy Control Group (*n*: 65)	*p* Value
Male sex, *n* (%)	37 (53.6)	32 (49.2)	0.61
Age, years, median (min–max), years	13 (6–18)	15 (5–17)	0.144
Height z-score	−0.04 (1.86)	0.14 (1.92)	0.708
Weight z-score	0.30 (1.31)	0.35 (1.24)	0.659
Smoking exposure, *n* (%)	18 (26.5)	14 (21.5)	0.501
R5 (kPa·L^−1^ s), Interquartile range	0.41 (0.20)	0.37 (0.18)	0.019
R20 (kPa·L^−1^ s), Interquartile range	0.25 (0.11)	0.25 (0.13)	0.956
R5–R20 (kPa·L^−1^ s), Interquartile range	0.140 (0.13)	0.10 (0.08)	0.001
X5 (kPa·L^−1^ s), Interquartile range	−0.10 (0.07)	−0.09 (0.07)	0.101
X20 (kPa·L^−1^ s), Interquartile range	0.02 (0.15)	0.01 (0.09)	0.570
AX (kPa·L^−1^), Interquartile range	1.05 (1.14)	0.86 (0.61)	0.035
Fres (Hz), Interquartile range	18.58 (4.79)	19.14 (8.22)	0.312

## Data Availability

The data presented in this study are available on request from the corresponding author.
